# Irreversible atrophy in memory brain regions over 7 years is predicted by glycemic control in type 2 diabetes without mild cognitive impairment

**DOI:** 10.3389/fnagi.2024.1367563

**Published:** 2024-03-25

**Authors:** Nádia Canário, Joana Crisóstomo, João Valente Duarte, Carolina Moreno, Hugo Quental, Leonor Gomes, Francisco Oliveira, Miguel Castelo-Branco

**Affiliations:** ^1^Coimbra Institute for Biomedical Imaging and Translational Research (CIBIT), Institute of Nuclear Sciences Applied to Health (ICNAS), University of Coimbra, Coimbra, Portugal; ^2^Faculty of Medicine, University of Coimbra, Coimbra, Portugal; ^3^Department of Endocrinology, Centro Hospitalar e Universitário de Coimbra (CHUC), Coimbra, Portugal; ^4^Champalimaud Research, Champalimaud Foundation, Lisbon, Portugal

**Keywords:** type 2 diabetes, glycemic control, grey matter, memory, parahippocampal complex, longitudinal

## Abstract

Memory-related impairments in type 2 diabetes may be mediated by insulin resistance and hyperglycemia. Previous cross-sectional studies have controversially suggested a relationship between metabolic control and a decrease in hippocampal volumes, but only longitudinal studies can test this hypothesis directly. We performed a longitudinal morphometric study to provide a direct test of a possible role of higher levels of glycated hemoglobin with long term brain structural integrity in key regions of the memory system – hippocampus, parahippocampal gyrus and fusiform gyrus. Grey matter volume was measured at two different times – baseline and after ~7 years. We found an association between higher initial levels of HbA_1C_ and grey matter volume loss in all three core memory regions, even in the absence of mild cognitive impairment. Importantly, these neural effects persisted in spite of the fact that patients had significantly improved their glycemic control. This suggests that early high levels of HbA1c might be irreversibly associated with subsequent long-term atrophy in the medial temporal cortex and that early intensive management is critical.

## Introduction

The parahippocampal complex seems to be a critical region in the pathophysiology of type 2 diabetes mellitus (T2DM), mild cognitive impairment (MCI), and dementia (Zilliox et al., 2016; Bello-Chavolla et al., 2019). Memory-related impairments are found in this population (Zilliox et al., 2016), along with evidence of a link between T2DM and MCI and/or dementia (Bello-Chavolla et al., 2019). Nonetheless, a clear understanding of the pathophysiological mechanisms in T2DM and the development of cognitive deterioration and ultimately dementia is still far from understood.

Pathophysiological mechanisms linking T2DM and Alzheimer’s dementia (AD) and brain atrophy have been suggested to involve insulin resistance and hyperglycemia (Bello-Chavolla et al., 2019; Li et al., 2021). However human studies investigating the role of metabolic control in brain atrophy are mainly cross-sectional, preventing a direct test of a temporal link between glycemic control and brain structural integrity (Gold et al., 2007; Moran et al., 2013; Hirabayashi et al., 2016; Ogama et al., 2018). Furthermore, longitudinal studies on brain atrophy so far were mostly dedicated to investigating total brain volume and/or hippocampus alone (Debette et al., 2011; Callisaya et al., 2019). In addition, no associations with hyperglycemia have been particularly explored. Knowing whether impaired metabolic control leads to irreversible brain atrophy in T2DM, especially in memory-related regions, is important for the implementation of an early and timely management of metabolic control.

In this study, we report a longitudinal study over 7 years to investigate whether initial higher levels of glycated hemoglobin (HbA_1C_) in T2DM, are associated with a long-term decrease in grey matter volume in three core brain regions involved in memory processing – hippocampus, parahippocampal gyrus, and fusiform gyrus and if these changes revert upon improvement of metabolic control.

## Materials and methods

### Participants

A total of 16 T2DM, patients and 13 non-diabetic controls were initially recruited for this study. Participants were selected from the DIAMARKER database (Duarte et al., 2015; Crisóstomo et al., 2021). Patients were diagnosed in the Endocrinology Department of the Coimbra University Hospital Center using standard World Health Organization criteria (WHO, 2011) and were selected to be part of the follow-up study based on the following criteria: (1) age between 45 and 75 years old; (2) not having a history of central nervous system disorder, neoplastic, inflammatory, or mental diseases and (3) no history or current alcohol abuse. Fasting blood samples were collected and all participants were screened for the presence of amnestic MCI (aMCI), at the follow-up visit, based on Petersen’s clinical criteria (Petersen, 2016), and performed further brief cognitive assessment (Table 1). aMCI is a subtype of MCI characterized by the presence of objective memory complaints along with an objective impairment in episodic memory without fulfilling accepted criteria for dementia (Dubois et al., 2007; Albert et al., 2011). The neuropsychological tests used for the aMCI screening were: (a) the subjective memory complaints questionnaire in order to assess the presence of memory complaints (Schmand et al., 1996; Ginó et al., 2008), the Montreal cognitive assessment for cognitive screening (Nasreddine et al., 2005; Freitas et al., 2011), the cued and selective reminding test for verbal episodic memory assessment (Lemos et al., 2015), the copy and delayed recollection of the Rey’s complex figure for perceptual organization and visual episodic memory assessment, respectively (Rocha and Coelho, 2002), and the similarities and matrix reasoning from the Wechsler Intelligence Scale III, for verbal and visual conceptualization skills assessment (Wechsler, 2008). We also delievered to participants the geriatric depressive scale (Yesavage et al., 1982; Barreto et al., 2008) to test for the presence of depressive symptoms and the older adults functional assessment inventory in order to screen for dementia status (Sousa et al., 2013). All neuropsychological tests in order to exclude MCI were versions that were previously translated and validated versions for the Portuguese population, which were not conducted by our team. Regarding the translation and validation of the neuropsychological tests used in this study, we recommend contacting the original authors or entities responsible for the initial translation and validation. The neuropsychological assessment was performed at the 2nd visit.

**Table 1:** Clinical and neuropsychological measures.

	T2DM [M (S.E)] *N* = 12	Controls [M (S.E)] *N* = 12	*p*	T2DM *p* (T1 vs. T2)	Controls *p* (T1 vs. T2)
Age (years)	62.4 (2.21)	59.5 (1.71)	–	–	–
Weight (kg)	81.77 (4.34)	75.55 (3.23)	–	–	–
Height (m^2^)	1.67 (0.03)	1.70 (0.03)	–	–	–
Duration of disease (years)	18.00 (2.22)	–	–	–	–
HbA_1C_ (%) T1	10.02 (0.94)	5.69 (0.13)	<0.002*	<0.021*	>0.742
HbA_1C_ (%) T2	8.08 (0.47)	5.67 (0.15)	<0.001*		
Glycose (mg/dL) T1	192.17 (25.79.35)	93.56 (4.12)	<0.004*	>0.151	>0.645
Glycose (mg/dL) T2	152.33 (21.68.)	95.17 (4.81)	<0.025*		
Total chol. (mg/dL) T1	187.50 (12.08)	198.20 (15.02)	>0.585	>0.082	>0.284
Total chol. (mg/dL) T2	163.42 (14.71)	180.09 (7.29)	<0.020*		
LDL (mg/dL) T1	131.17 (10.86)	137.50 (11.71)	>0.695	<0.027*	>0.120
LDL (mg/dL) T2	97.00 (15.85)	110.25 (8.22)	>0.073		
Triglycerides (mg/dL) T1	181.67 (30.337)	100.70 (15.49)	<0.031*	>0.157	>0.720
Triglycerides (mg/dL) T2	144.75 (39.896)	116.37 (21.66)	>0.311		
Med – oral AntiD (%)	58	–	–	–	–
Med – Insulin (%)	75	–	–	–	–
Smok. habits (Yes; No)	2;7	0;11	–	–	–
Quit (Yes)	3	1	–	–	–
Alcoh. habits (Yes; No) (Yes; No)	6;5	5;7	–	–	–
Quit (Yes)	1	0	–	–	–
Exerc. habits (Yes; No) (Yes;No)	7;5	–	–	–	–
SMC	1.92 (0.61)	2.25 (0.63)	>0.615	–	–
GDS-30	6.67 (0.99)	4.75 (1.29)	>0.171	–	–
FCSRT					
Total immediate recall	43.25 (1.22)	43.17 (1.22)	>0.292	–	–
Total delayed recall	15.25 (0.31)	15.08 (0.29)		–	–
Free recall efficacy	0.60 (0.04)	0.67 (0.03)		–	–
Toral recall efficacy	0.90 (0.25)	0.90 (0.02)		–	–
RCF	–				
Copy	31.2 (0.96)	32.92 (0.61)	>0.241	–	–
Immediate Recall	17.95 (1.89)	18.38 (1.99)		–	–
Delayed Recall	18.71 (2.08)	18.71 (1.90)		–	–
IAFAI	–				
Cognitive incapacity (%)	0.17 (0.17)	0	>0.118	–	–
Emotional incapacity (%)	0	0		–	–
Physical incapacity	1.41 (0.60)	0.17 (0.17)		–	–
MoCA (total score)	23.42 (1.04)	26.50 (0.88)	>0.703	–	–
Matrices (WAIS-III)	13.17 (1.74)	17.83 (0.88)	>0.824	–	–
Similarities (WAIS-III)	19.00 (1.82)	25.17 (1.44)	>0.812	–	–

Sample characterization by mean (M) and standard errors (SE). Displays clinical and neuropsychological measures.T2DM, type 2 diabetes mellitus; T1, time 1 (1st visit); T2, time 2 (2nd visit); y, years; AntiD, antidiabetics; SMC, subjective memory complaints; GDS-30, geriatric depression scale; FCSRT, free and cued selective reminding test; RCF, rey-osterrieth complex figure; IAFAI, functional assessment inventory of adults and elderly; MoCA, montreal cognitive assessment. *Significant at 0.05 level. Age, weight and height depicted for the second visit.

Overall, 5 participants were excluded from the sample due to aMCI (3 participants with T2DM and 1 from the control group) and due to abdominal perimeter incompatible with MRI measures (1 participant with T2DM), making a total of 12 T2DM patients and 12 controls (Table 1). The participants included in the analysis did not meet criteria for aMCI. Both groups had an 8/4 male/female ratio. The average number of years between visits was 7 years, ranging from 5 to 9.

All participants provided written informed consent. The present study complied with the Declaration of Helsinki and was approved by the Ethics Committee of the Faculty of Medicine, University of Coimbra.

### Image acquisition

Magnetic resonance imaging (MRI) data were acquired on a 3-Tesla Prisma scanner (upgraded from an initial Tim Trio version, as well as the 12-channel birdcage to a 64-channel head coil). The 3D MPRAGE scans were acquired using a T1w gradient echo pulse, with a flip angle of 7°, a repetition time (TR) of 2.530 milliseconds, echo time (TE) of 3.42 milliseconds, and an inversion time (TI) of 1.100 milliseconds. We acquired a total of 176 slices per structural scan, yielding whole-brain coverage with a voxel size of 1 × 1 × 1 mm^3^ and a field of view (FOV) of 256 mm.

### MRI data processing and statistical analysis

T1-weighted images were processed using the computational anatomy toolbox 12 (CAT12), implemented within the statistical parametric mapping 12 (SPM12), running in MATLAB version R2017b (The Mathworks, MA, United States).

All images for voxel based morphometry (VBM) were segmented using the segmentation routine for longitudinal designs implemented in CAT12. The segmented scans were spatially normalized according to diffeomorphic anatomic registration through exponentiated Lie algebra algorithm (DARTEL), and registered to the Montreal neurological institute (MNI) template. An 8 mm full width at half maximum (FWHM) Gaussian filter was applied to spatially smooth the images. We further computed, for each participant, new images containing differences in grey matter volume by subtracting the images of time 1 (T1) from the images of time 2 (T2). Each new image was corrected voxel-wise for the respective total intracranial volume (TIV), using the following computation: $\left(\frac{T2}{TIV}\right)-\left(\frac{T1}{TIV}\right)$ . We then projected the normalized grey matter maps to the automatic anatomical labeling (AAL) atlas and quantified the differences in grey matter volume in three different regions of interest (ROIs) in both hemispheres – hippocampus, parahippocampal gyrus, and fusiform gyrus - to correlate with baseline HbA_1C_ levels. To investigate changes across time we also studied the association between longitudinal changes of HbA_1C_ levels and longitudinal changes in grey matter. Because there are variables whose distribuition failed to pass the normality test assessed by the Shapiro–Wilk test, all correlations were performed using a two-tailed non-parametric partial correlation test, controlled for education. Since patients had significantly improved their levels of HbA_1C_ between the two time points we also computed non-parametric partial correlations between longitudinal changes in HbA_1C_ and the longitudinal measures of grey matter volume, with an additional correction for the baseline levels of HbA_1C._ Between-group comparisons were performed using the Mann–Whitney U test. A statistical significance of 0.05 was applied.

We further complemented our analysis by performing a whole-brain VBM linear regression analysis between the HbA_1C_ at baseline and the differences in grey matter volume to achieve a better characterization of regional differences without the atlas-based rigid locations’ constraints. Only the results in the referred ROIs and nearby areas were considered, given our hypothesis driven approach. Nonetheless, it also gave information about broad changes in grey matter volume in association with HbA_1C_ levels at baseline. Statistical VBM maps were obtained using a *p* < 0.008, corrected by the total number of 6 target ROIs. An additional correction at a cluster level was also implemented (k = 142 voxels). The linear regression model was also corrected for participants’ education. It was not necessary to control the analysis for age because the groups were age-matched (see Table 1 for descriptive statistics).

Sample characterization regarding all clinical variables was performed by two-tailed independent sample t-tests or the nonparamnetric analogue – Mann–Whitney U-test – for group comparison. Two-tailed paired sample *t*-tests or the wilkoxon rank-sum test were performed for within-group comparisons. An ANCOVA controlling for education was performed for the neuropsychological all measures with only one dependent variable. The between-group comparison for the remaining neuropsycological tests with more than one dependent variable was tested by using a nonparametric multivariate test (MANOVA). The choice for a nonparametric statistical approach was done for all testing whose data were not normally distributed, assessed by the Shapiro–Wilk test.

Descriptive and inferential statistics were performed on IBM SPSS statistical package (v.27.0.1).

## Results

**Figure 1:**
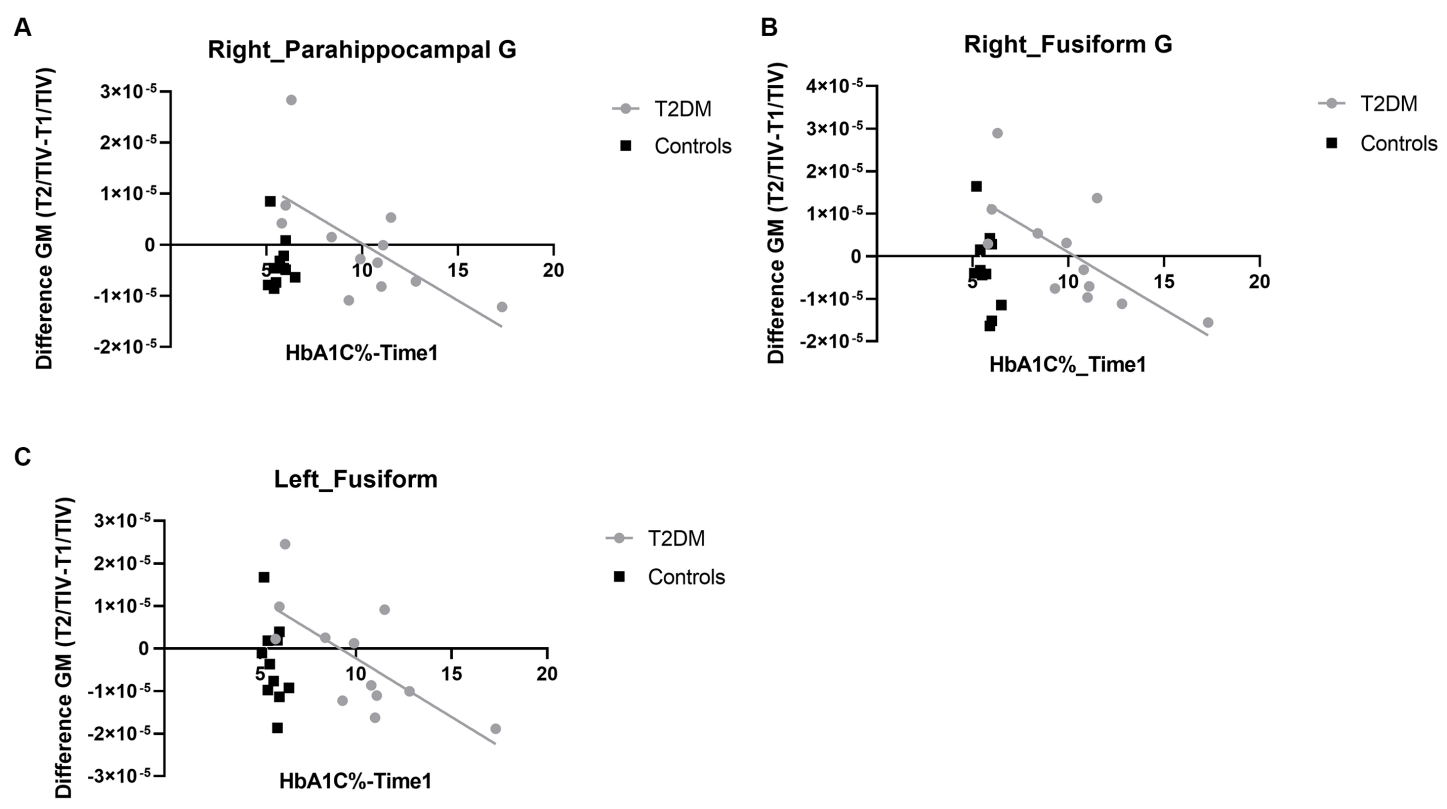
Linear regression plots depicting the association between the longitudinal differences in grey matter volume and HbA_1C_ levels. **(A)** Depicts the linear regression plot in right parahippocampal gyrus. **(B)** The linear regression plot in right fusiform gyrus and **(C)** depicts the same analysis in the homologue hemisphere. G, gyrus; T2DM, type 2 diabetes mellitus; GM, grey matter. Note that values in Y axes are normalized to total intracranial volume, explaining their magnitude and rendering them unitless.

We found a negative statistically significant correlation between baseline HbA_1C_ and the longitudinal changes in grey matter volume in the T2DM group in three ROIs – the right parahippocampal gyrus (*r*s = −0.665, *p* = 0.026, *n* = 12, power = 0.889), the left fusiform gyrus (*r*s = −0.703, *p* = 0.016, *n* = 12, power = 0.937) and the right fusiform gyrus (*r*s = −0.689, *p* = 0.019, *n* = 12, power = 0.921; see Figure 1). We also found a marginal negative correlation between HbA_1C_ and the changes in grey matter volume in the right hippocampus (*r*s = −0.580, *p* = 0.062, *n* = 12, power = 0.742). No statistically significant correlation was found for the left hippocampus (*r*s = −0.446, *p* = 0.149, *n* = 12) and the left parahippocampal gyrus (*r*s = −0.495, *p* = 0.121, *n* = 12) in the T2DM, group. As expected, we found no statistically significant correlations between the control group grey matter volume and HbA_1C_ in any of the ROIs. Importantly, no correlation was found in T2DM between the longitudinal changes in HbA_1C_ and the longitudinal changes in grey matter volume of the regions whose grey matter volume which proved to be initially correlated with HbA1_c_, showing that the improvement in metabolic control was not sufficient to revert changes in grey mattern integrity. This result is also corroborated by whole-brain VBM analysis.

**Figure 2:**
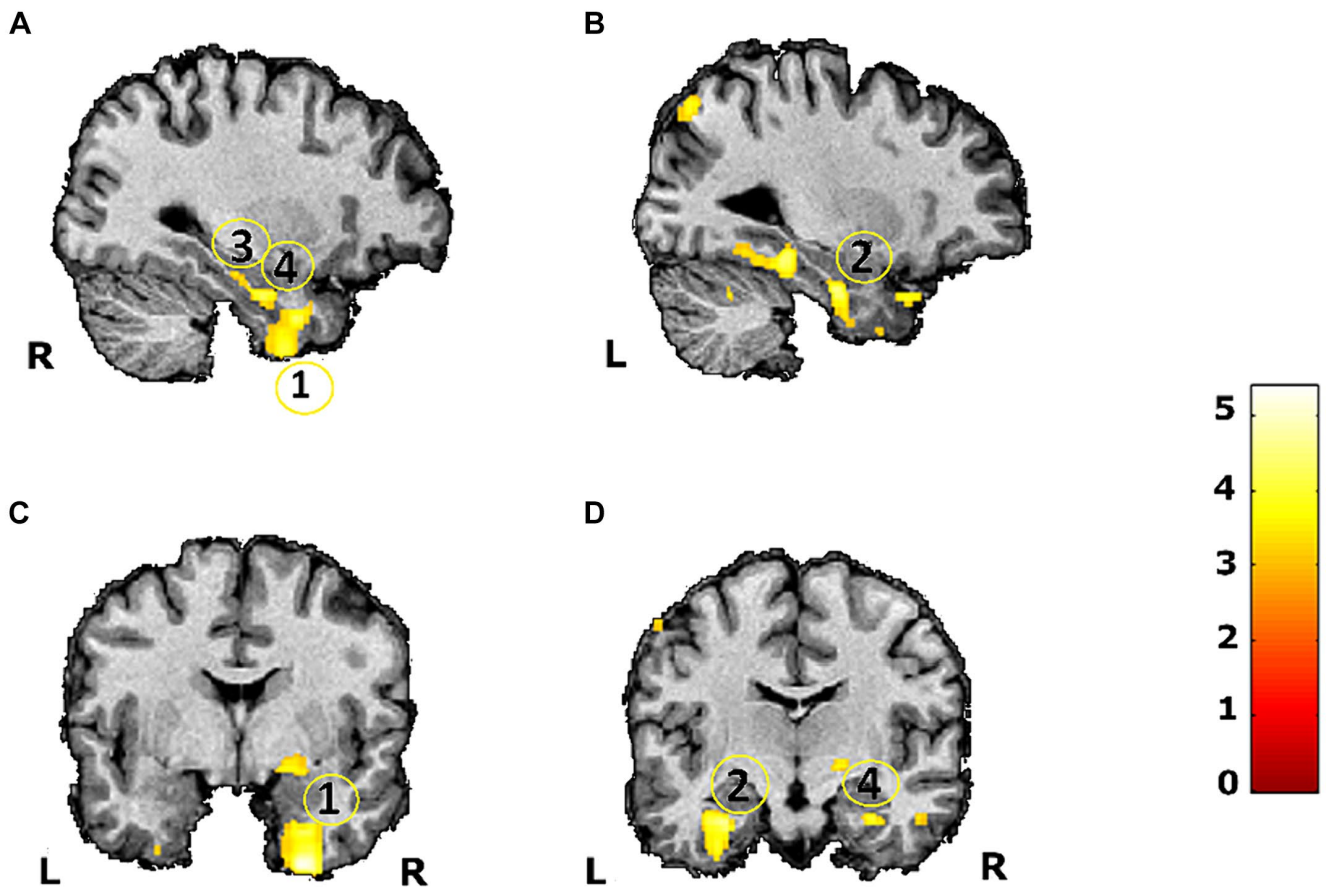
VBM linear regression whole brain (replication of ROI analysis) maps between the HbA_1C_ at baseline and differences in grey matter volume assessed during a 7-year follow-up study in 12 T2DM patients (*p* < 0.008). Scale represents *t* values. (1) Right inferior temporal gyrus (including voxels in right parahippocampal gyrus and right fusiform gyrus) **(A,C)**; (2) Left parahippocampal gyrus (including voxels in left fusiform gyrus) **(B,D)**; (3) Right parahippocampal gyrus **(A)**; (4) Right hippocampus **(A,D)**. L, left; R, right.

We were able to replicate the ROI approach by using whole-brain VBM linear regression analysis. We found for the T2DM, group three different main clusters in the regions under study showing a longitudinal negative correlation between changes in grey matter volume and the HbA_1C_ at baseline: (a) one cluster, with 1,143 voxels (26, −2, −50), whose peak voxel was in the right inferior temporal gyrus (rITG) and included voxels in right fusiform gyrus and right parahippocampal gyrus; (b) a cluster, composed of 544 voxels (32, −11, −27), with peak voxel on the right parahippocampal gyrus and (c) another cluster, with 598 voxels, on left parahippocampal gyrus (−29, −14, −30). This latter cluster had also voxels in the left fusiform gyrus, being in congruence with our ROI analysis taken from the AAL atlas, which independently showed a correlation between HbA_1C_ and changes in grey matter volume in left fusiform gyrus (see Figure 2). Importantly, the more anterior part of the right parahippocampal gyrus cluster included voxels in right hippocampus. This was not the case for the left parahippocampal gyrus. This analysis provides statistical support even for the right hippocampus for the ROI analysis taken from the AAL atlas. Additionally, the cluster from the whole-brain VBM analysis revealed voxels in left parahippocampal gyrus. No suprathreshold clusters were found for the control group in the whole-brain linear regression analysis. The description of other regions, obtained for the linear regression analysis for the T2DM group, are depicted in Supplementary Table S1.

## Discussion

This study provides evidence of a negative association between grey matter volume loss along ~7 years in medial temporal lobe memory encoding regions (right hippocampus, both parahippocampal gyrus and both FGs), and initial levels of HbA_1C_ in a T2DM, sample without MCI. This suggests that impaired glycemic control (higher levels of HbA_1C_) might lead to long term atrophy in memory related regions. Importantly, longitudinal changes in HbA_1C_ did not impact longitudinal modifications in grey volume suggesting that the atrophy found in these regions might be irreversible. This demonstrates the importance of early and timely glycemic control to prevent such irreversible changes. The ideia that irreversible changes in brain morphometrical properties might occur in T2DM in relation with hyperglycemia is corroborated by studies that focused on the complex deleterious effect of hyperglycemia in the brain. Possible mechanisms behind brain atrophy may include effects in addition to direct vascular impairments (Williams et al., 1998; Biessels et al., 2006; Moulton et al., 2015). We suggest that subtle alterations in neurovascular coupling is indeed suggested for people with T2DM (Duarte et al., 2015, 2023) or even direct damage to neurons that can also occur prior to vascular damage (Reis et al., 2014) may play a role. The study by Reis et al. (2014) indeed found that both people with type 1 diabetes, with and without blood-retinal barrier’s dysfuntion, sustained significant differences in visual functions suggesting that neuronal changes in retina occur prior to vasculopathy. Furthermore, augmented oxidative stress, causing inflammation is also another mechanisms behind brain atrophy. Dysfunctional insulin signaling and elevated blood glucose levels lead to heightened expression and activation of NF-κB, a pivotal participant in the inflammatory cascade and a regulator of apoptosis (Sima et al., 2009; Moulton et al., 2015). Moreover, advanced glycation end products, also leads to inflammation, as a consequence of chronic activation of microglia, leading to neuronal and glial apoptosis (Moulton et al., 2015; Sun et al., 2020; Yu et al., 2023). We favour the view that neurodegenerative processes underlying T2DM’s pathophysiology might also leads to the presence of brain atrophy, where toxic glucose levels have a harmful impact on various biochemical processes associated with tau integrity, promoting tau cleavage and phosphorylation (Kubis-Kubiak et al., 2019; Huang et al., 2020). Additionally, elevated glucose levels influence the production and degradation of Aβ (Macauley et al., 2015; Kubis-Kubiak et al., 2019). There is evidence indicating that certain Aβ products undergo predominant degradation through the proteasome pathway, which is significantly destabilized by increased glucose levels (Kubis-Kubiak et al., 2019; Patil et al., 2020). Nonetheless, different regions seem to have specific vulnerabilities and different mechanisms might underly atrophy in particular regions.

Human studies investigating the role of metabolic control in brain structural dysfunction are overall cross-sectional. In Ogama et al. (2018), postprandial hyperglycemia was associated with lower brain volume. Another study found that 2-h postload glucose levels were associated with lower total brain volume and hippocampus volume (Hirabayashi et al., 2016). Studies with animal models have found an association between hyperglycemia and memory loss in relation with signs of neuroinflammation (Wanrooy et al., 2018; Rom et al., 2019) namely in the hippocampus (Wanrooy et al., 2018). Several studies focused on the association between hyperglycemia, oxidative stress, and brain atrophy (Ogama et al., 2018), and others investigated the effect of hyperglycemia in microvascular structures (Stehouwer, 2018). Indeed, brain structures are fairly vulnerable to microvascular disturbances since brain microvessels have low resistance and sensitive auto-regulatory processes, being more vulnerable to phenomena like augmented pulsatile load that occurs with elevated glucose levels (Stehouwer, 2018). Nonetheless, a recent study suggested that brain atrophy in hippocampal subfields are associated with cognitive impairment in this disease rather than microstructural lesions, where HbA_1c_ levels were negatively correlated with hippocampal atrophy but not with the burden of white matter hyperintensities (Zhang et al., 2021). Furthermore, previous randomized clinical trial testing the effects of intensive glycemic control in T2DM participants versus a standard glycemic control on the longitudinal changes in brain atrophy showed that intensive glycemic treatment decelerated the volume loss in regions like bilateral superior temporal gyrus, left pre and postcentral gyri, in bilateral fronto-orbital gyrus and bilateral frontal cingulate regions (Erus et al., 2015). Importantly, no significant changes were found in medial temporal gyrus and in particular in hippocampus and parahippocampal regions (Erus et al., 2015) which is in agreement with our study.

In overall, several possible mechanisms might explain the association between hyperglycemia and an augmented loss of brain volume inT2DM, from microvascular complications to augmented toxicity and neuroinflamation, leading to increased dysfunction. This is the first longitudinal study to provide evidence for a possible irreversible long term loss in a T2DM, sample without MCI. The association between long term brain atrophy and early HbA_1C_ changes in core regions of the inferior and medial temporal lobes, such hippocampus and parahippocampal gyrus, is of particular interest given the hypothesized association between T2DM, and AD (Bello-Chavolla et al., 2019). Evidence that initially impaired metabolic control in T2DM, patients with no MCI may be associated with irremediable long term brain atrophy in key structures in the medial temporal lobe involved in memory processes observed within 7 years, consolidates the idea that pathological events precede cognitive deterioration. Furthermore, sex differences have been suggested to underlie cognitive deterioration in T2DM, with female having higher risk for accelerated cognitive deterioration (Verhagen et al., 2022). This variable was matched across our groups.

The present study provides evidence for a possible irreversible deleterious effect of poor glycemic control and the loss of grey matter volume in participants with T2DM, without mild cognitive impairment. Since participants neither fulfilled criteria for aMCI nor showed different cognitive profile relative to controls in any cognitive measures we suggest that structural atrophy might proceded cognitive deterioration. Further monitoring of the cognitive status of these participants is needed to explore if the consequences of poor glycemic control on the longitudinal changes in structural integrity in memory-related regions have a deleterius effect in cognition. The results highlight the importance of an early intensive control of glycemia and overall intensive management of T2DM.

## Data availability statement

The raw data supporting the conclusions of this article will be made available by the authors, without undue reservation.

## Ethics statement

The studies involving humans were approved by Comissão de Ética da Faculdade de Medicina da Universidade de Coimbra. The studies were conducted in accordance with the local legislation and institutional requirements. The participants provided their written informed consent to participate in this study.

## Author contributions

NC: Conceptualization, Formal analysis, Investigation, Writing – original draft. JC: Investigation, Writing – review & editing. JD: Investigation, Methodology, Writing – review & editing. CM: Investigation, Validation, Writing – review & editing. HQ: Investigation, Writing – review & editing. LG: Investigation, Validation, Writing – review & editing. FO: Investigation, Supervision, Writing – review & editing. MC-B: Conceptualization, Data curation, Funding acquisition, Investigation, Project administration, Resources, Supervision, Validation, Writing – review & editing.
