# Supplementary material for: Irreversible atrophy in memory brain regions over 7 years is predicted by glycemic control in type 2 diabetes without mild cognitive impairment
Source: Front Aging Neurosci. 2024 Mar 25;16:1367563. doi: 10.3389/fnagi.2024.1367563 (PMC10999637; doi:10.3389/fnagi.2024.1367563)
Supplement: Supplementary file 1 [file Table_1.DOCX]

|  | **MNI coordinates** | | |  |
| --- | --- | --- | --- | --- |
| **Region** | **X** | **Y** | **Z** | **Nr Voxels** |
| Left Extrastriate Visual Cortex (BA18) | -15 | -99 | -9 | 3698 |
| Right Extrastriate Visual Cortex (BA18) | 15 | -99 | -9 | 292 |
| Right Caudate | 21 | 5 | -11 | 530 |
| Right Primary Visual Cortex (BA17) | 18 | -66 | -8 | 904 |
| Left Prefrontal Cortex (BA9) | -6 | 26 | -21 | 199 |
| Right Inferior Frontal Lobe (BA44) | 47 | 30 | -12 | 185 |
| Left Angular Gyrus (BA39) | -47 | -57 | 41 | 330 |
| Left Posterior Cingulate Cortex (BA23) | -54 | -24 | -29 | 205 |
| Left Middle Temporal Gyrus (BA21) | 51 | -41 | 2 | 161 |
| Superior Parietal Cortex (BA7) | -27 | -77 | 48 | 194 |
| Left Temporal Pole (BA38) | -30 | 15 | -33 | 147 |
| Right Fusiform Gyrus (BA37) | 53 | -53 | -17 | 210 |

**Supplementary table 1:** Whole-brain regions obtained from the linear regression analysis performed for the T2D group. Results were obtained from a negative correlation between baseline levels of HbA_1C_ and the longitudinal changes in grey matter volume.
